# Synthesis and characterisation of cross-linked chitosan composites functionalised with silver and gold nanoparticles for antimicrobial applications

**DOI:** 10.1080/14686996.2017.1344929

**Published:** 2017-07-20

**Authors:** Catherine Ryan, Emma Alcock, Finbarr Buttimer, Michael Schmidt, David Clarke, Martyn Pemble, Maria Bardosova

**Affiliations:** ^a^ Micro & Nano Systems Centre, Tyndall National Institute, University College Cork, Cork, Ireland; ^b^ Department of Chemistry, University College Cork, Cork, Ireland; ^c^ Department of Microbiology & Alimentary Pharmabiotic Centre, University College Cork, Cork, Ireland; ^d^ Faculty of Electrical Engineering and Information Technology, Slovak Technical University in Bratislava (STUBA), Bratislava, Slovak Republic

**Keywords:** Chitosan, cross-linked, interpenetrating polymer network, composite, silver nanoparticle, gold nanoparticle, antimicrobial, 30 Bio-inspired and biomedical materials, 103 Composites, 301 Chemical syntheses / processing, 503 TEM, STEM, SEM, 505 Optical / Molecular spectroscopy, 600 Others: Surface and mechanical analysis, Antimicrobial tests

## Abstract

We present a study of a range of cross-linked chitosan composites with potential antimicrobial applications. They were formed by cross-linking chitosan and siloxane networks and by introducing silver and gold nanoparticles (NPs). The aim was to investigate whether adding the metal NPs to the chitosan-siloxane composite would lead to a material with enhanced antimicrobial ability as compared to chitosan itself. The composites were synthesised in hydrogel form with the metal NPs embedded in the cross-linked chitosan network. Spectroscopic and microscopic techniques were employed to investigate the structural properties of the composite and the tensile strength of the structures was measured. It was found that the addition of metal NPs did not influence the mechanical strength of the composite. A crystal violet attachment assay results displayed a significant reduction in the attachment of *E. coli* to the cross-linked chitosan surfaces. Release profile tests suggest that the metal NPs do not contribute to the overall antimicrobial activity under neutral conditions. The contribution to the mechanical and antimicrobial properties from cross-linking with siloxane is significant, giving rise to a versatile, durable, antimicrobial material suitable for thin film formation, wound dressings or the coating of various surfaces where robustness and antimicrobial control are required.

## Graphical abstract

**Figure:**
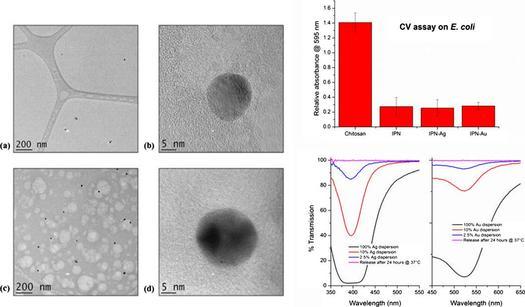

## 1. Introduction

### 1.1. Chitosan

Chitosan has many uses in a variety of industries, specifically in the biomedical field. It has significantly surpassed the usage limits of its predecessor, chitin [1]. Chitin’s disadvantages stem from its distinct lack of ease of processing, due to its insolubility in most common solvents. This has been attributed to the strong intra- and inter-polymer hydrogen bonds formed by the acetamido functionality; these strong bonds contribute greatly to the insolubility of the molecule. Although soluble in harsh solvents such as hexafluoroisopropyl alcohol, common practice is to avoid use of these solvents due to their associated hazards, especially at batch-scale in industry [2]. The conversion to chitosan is a relatively simple one, involving the deacetylation of the acetamido group to yield an amino group. This functional group interconversion confers more favourable physical properties, for example it becomes soluble in acidic solution due to the presence of proton-sensitive amino groups, allowing dissolution in media of pH less than 6 [3]. Within this process, a factor to be considered is the threshold of deacetylation required to distinguish chitin from chitosan. In general, chitin is said to have a degree of acetylation of approximately 90%, meaning the presence of some acetyl groups within the polymer is unavoidable [4]. When deacetylation reaches approximately 50%, the polymer is regarded as chitosan [5]. Therefore, chitosan is essentially a copolymer composed of randomly distributed repeating units of both *N*-acetyl-D-glucosamine and *N*-D-glucosamine, linked through β-(1–4)-glycosidic bonds [6]. Advantageously, the desirable attributes of chitin, such as its biological and ecological compatibility, are unaffected by the conversion to chitosan. It is the innate physical and chemical properties possessed by chitosan which contribute to the vast array of potential applications within the biomedical field for this material that are currently under investigation [7].

Varying molecular weight and degree of deacetylation are the two key characteristics which influence the properties and functionality of chitosan. Properties such as mechanical strength, thermal stability, permeability [6,8], swelling ability [9] and pH-sensitivity [10] are greatly affected by variations in the two key characteristics. Biological applications of chitosan rely on manipulation of some of these key reactivity and structural features [11]. Drug delivery is a prominent area in chitosan research – an application which exploits the biocompatibility and pH-sensitivity of the polymer [12]. Chitosan has also been proven to demonstrate diverse antimicrobial capabilities, acting as a biocidal agent against fungi [13] and both Gram-positive and Gram-negative bacteria [14]. The presence of both a primary and secondary alcohol at the C-2 and C-6 positions of each monomeric unit, coupled with a reactive amine at C-3 in the deacetylated monomers, show a key structure activity relationship [3]. In an era of extensive concern regarding antibiotic resistance, the emergence of chitosan’s antimicrobial potential is one of considerable interest. At the interface of materials and biomedical science, the precise mechanism of action of chitosan as an effective antimicrobial agent is unknown, but studies have suggested various explanations. One rationale, suggested by Li et al., attributes antimicrobial activity to the amine functional group [15]. At low pH, it has the potential to become ionised to NH_3_^+^, introducing the possibility of electrostatic interactions with negatively charged microbial cell walls. It is thought this alters the permeability and strength of the cell wall, allowing for weakening and eventual rupture, thus killing the organism [15]. This activity is seen to cease at pH values greater than 6, as chitosan loses its charge, and oftentimes, can be seen precipitating from the solution [16]. The molecular weight of chitosan is also shown to influence antibacterial activity. High molecular weight chitosan is regarded as too large to permeate the cell walls of bacteria. It is thought to cluster on bacterial cell walls, inhibiting entry of essential nutrients and oxygen, inevitably resulting in cell death [17]. In contrast, low molecular weight chitosan possesses the ability to penetrate cells, where it is suspected of binding to cell DNA, prohibiting mRNA synthesis and resulting in the termination of cell multiplication [18]. A study conducted by Liu et al. described the influence of chitosan concentration and molecular weight on its effectiveness as an antimicrobial agent against *Escherichia coli* (*E. coli*). At low concentration, it was concluded that chitosan displayed no biocidal effect – indeed was sometimes seen to promote bacterial growth – but at higher concentrations it acted as an effective agent, resulting in cell death [14]. Agglutination of cells resulting in cell death is the mechanism of action proposed. For this to occur, a threshold concentration of chitosan is required. Below this, bacteria continue to multiply and thrive [14]. The degree of deacetylation must also be considered when discussing the antimicrobial activity of chitosan. Chitosan with a high degree of deacetylation (HDD) shows a higher antimicrobial efficacy. HDD chitosan shows a higher acid solubility and so is expected to demonstrate better antimicrobial potential than chitosan with a low degree deacetylation (LDD). Lysozyme, a biological enzyme responsible for cell lysis demonstrates degradative action towards chitosan. Lysozyme is far more efficient at cleaving the bond in *N*-acetyl glucosamine, which is more abundant in LDD chitosan, than the corresponding bond in *N*-glucosamine. For this reason, HDD chitosan is seen as a more robust polymer [9].

### 1.2. Cross-linking

Although regarded as a highly applicable polymer, chitosan does possess some drawbacks, mainly due to its high aqueous solubility – a property which proves problematical in applications involving aqueous biological media. This issue can be overcome by the introduction of a cross-linker. A cross-link is formed through a chemical reaction, which links two polymers together, either through covalent/ionic bonds or weaker bonding interactions, for example, van der Waals forces [19]. The individual polymers within the cross-linked composite may together show new properties, whilst still maintaining their own critical features. One type of cross-link, which has undergone significant study, is the formation of an interpenetrating polymer network (IPN). According to the International Union of Pure and Applied Chemistry, an IPN can be defined as ‘a polymer comprising of two or more networks which are at least partially interlaced on a molecular scale but not covalently bonded to each other and so cannot be broken unless chemical bonds are broken’ [20]. Cross-linkers come in the form of polymers [21], oxides [22], metals [23] and amino acids [24], among other chemical entities. For the purpose of our research we synthesised and investigated a physically cross-linked network of two polymers, chitosan and siloxane, as well as incorporating metal nanoparticles (NPs) as further structure enhancers. There are many possible polymers which could successfully form an IPN with chitosan, especially due to the availability of chitosan’s functional groups for interaction. These include polymers containing carboxylic acids, epoxides and alcohols. However, the use of some of these polymers can be unfavourable as they may disrupt membrane formation [25]. One IPN that has been established as non-problematic in this regard is the IPN formed between chitosan and tetraethyl orthosilicate (TEOS) [26]. The chitosan-TEOS IPN (Chi-TEOS IPN) contains two main components: chitosan, which forms individual polymer chains, and TEOS, which undergoes hydrolysis followed by condensation to generate a siloxane polymer chain. These two chains subsequently cross-link to form a so-called Chi-TEOS IPN. Physical cross-linking takes place resulting in the individual polymer chains entangling and interlacing, held together by hydrogen bonds and van der Waals forces [27]. This combination results in the formation of an IPN which is flexible, due to the presence of chitosan while also being mechanically strong and insoluble in common aqueous systems, attributed to the siloxane cross-linker providing a structural backbone to the IPN [10]. This phenomenon was examined in a previous study from our laboratories in which tensile strength tests were carried out before and after cross-linking [28]. Plain chitosan exhibited elastic behaviour, tearing continuously under much less strain in comparison to the cross-linked Chi-TEOS IPN which demonstrated better mechanical strength, with sudden fracture of the membrane occurring at much higher stress levels. The reduced solubility and improvement in mechanical strength induced by cross-linking with TEOS is desirable in developing chitosan membranes for targeted applications, such as wound dressings. The effect of introducing colloidal particles (silica and polystyrene particles at a constant concentration and particle diameter in the range of 200–400 nm) was also examined in our previous study with results displaying a significant increase in tensile strength as a function of decreasing particle diameter, effectively increasing surface area. In this present study, the effect of incorporating metal NPs will be investigated in order to determine whether the tensile strength of the Chi-TEOS IPN is enhanced or degraded by the presence of metal NPs.

### 1.3. Silver nanoparticles

Accounting for approximately 25% of papers devoted to metal applications within biomedicine [29], silver is known to possess both anti-inflammatory and antimicrobial capabilities [30]. Seen as the most effective metallic antibacterial agent [31], with a higher potency than metals such as lead, tin, copper and chromium [32], silver has a diverse range of applications. These include medical implants, wound dressings, biosensors and emerging applications within the field of cancer therapeutics [33]. Recently, the use of silver nanoparticles (Ag NPs) has been seen to be more beneficial than the use of bulk silver within devices [32]. This has been attributed to the larger surface area and reduced mass of silver necessary to elicit an antimicrobial response. Because of this, Ag NPs are seen to possess a lower toxicity, making them a more favourable option. Recent studies have concluded that Ag NPs are more potent antibacterial agents than certain commercially available antibiotics [30].

An emerging explanation of the mechanism of bacterial resistance to these various agents involves the formation of biofilms. Biofilms can be defined as ‘microbial consortia embedded in self-produced exopolymer matrices composed of mainly exopolysaccharides’ [34]. A medical nuisance, biofilms are resistant to many known antibiotics, detergents and disinfectants. Usually, treatment of biofilms involves their physical removal, which is traumatic for patients [35]. Advantageously, Ag NPs have been seen to be capable of inhibiting biofilm formation, making them a potential alternative to commercial antibiotics in the treatment of biofilms [34]. The mechanism of antibacterial action of Ag NPs is thought to rely on surface interactions between silver and the bacterial cell wall. Due to the large surface area of Ag NPs, they are capable of anchoring to bacterial cell walls where they can stimulate changes in the strength and permeability of the cell. These changes can ultimately lead to cell lysis and extrusion of cell contents, resulting in bacterial cell death [36]. This mechanism of action was seen to be dependent on the type of bacterial cell being targeted. For example, according to Kim et al., Gram-negative *E. coli* shows a higher sensitivity to Ag NPs than Gram-positive *Staphylococcus aureus (S. aureus)* strains [37]. This difference can be rationalised by considering the structural differences between both bacteria. Gram-positive bacteria have much thicker cell walls made up of multiple peptidoglycan layers compared to a single peptidoglycan layer in the cell wall of Gram-negative bacteria [38]. The additional teichoic acid and peptidoglycan layers give extra protection to Gram-positive bacteria [33]. It has also been speculated that interactions between silver and lipid molecules in the cell membrane contribute to antibacterial activity. Silver is assumed to alter the fluidity of cell membranes through alterations in fatty acid composition. This can result in the loss of membrane integrity, allowing for easier penetration of Ag^+^ ions to the intercellular bacterial environment [39]. Thiol functional groups are present in many bacterial enzymes necessary for cell function. Ag^+^ ions can interact with sulfur atoms within the bacterial enzymes, disrupting their activity, with cell death an eventuality [40]. The extent of this interaction is also seen to rely on the composition of the bacterial cell wall. In order to cause an antibacterial effect, Ag^+^ ions must penetrate the cell wall and enter the cell cytoplasm. This has been shown to be more difficult in Gram-positive bacteria [33]. Durán et al. concluded that thiol-silver complexation resulted in the disruption of oxidative phosphorylation, a key step in metabolism, eventually leading to bacterial mortality [41]. Conflicting theories exist as to which form of silver is responsible for its antimicrobial potency. There is considerable evidence that ionic silver, Ag^+^, is responsible for the major antibacterial pathways, with many researchers believing that the silver cation has a high affinity for negatively charged DNA and thiol groups within bacteria. A study conducted by Xiu et al. found that under anaerobic conditions Ag NPs displayed no antibacterial activity [42]. This would suggest that antibacterial activity is solely due to Ag^+^. Many suggest the role of Ag NPs is simply to generate a continuous flow of Ag^+^ through oxidation of Ag^0^. It is believed these ions are then transported to their biological targets, where they elicit an antibacterial response [43].

### 1.4. Gold nanoparticles

In comparison to silver, gold is a metal which is not so commonly sought after for its antimicrobial abilities. In a review by Zhang et al. [44] it was proposed that gold, in both NP and ionic form, does not exhibit antimicrobial properties unless found at very high concentrations or in ionic complexes. Gold NPs (Au NPs) are generally stabilised by coating them with surfactant molecules such as polyvinylpyrrolidone (PVP) or sodium dodecyl sulfate (SDS) or ions such as citrate, which prevents agglomeration of the NPs in solution. In a study by Mukha et al. surfactant-stabilised Au NPs in the diameter range of 20–30 nm showed no antimicrobial activity against either *S. aureus* or *E. coli* [45]. In a similar test by Hernandez-Sierra et al., positive results were observed as the concentration of Au NPs in solution increased [46]. Interestingly, with the development of a ‘green’ synthesis approach to Au NPs there has been an improvement in results relating to bactericidal studies. ‘Green’ synthesis is essentially the name given to an eco-friendly approach to the chemical processing of Au NPs, which can be carried out using naturally occurring substances as reducing agents, such as flower extracts [47] or fungi [48]. Mishra et al. synthesised gold and silver NPs by a ‘green’ method with high antimicrobial efficiency observed against *S. aureus* [49]. The exact reasoning as to why there is such a marked improvement in results when synthesising Au NPs using the ‘green’ approach is thus far unknown but it is proposed it may be a synergistic effect enhanced by inherent bactericidal activity of the natural sources [50]. A similar dilemma arises in investigating the bactericidal properties of gold as it does with silver, namely, is the antibacterial activity observed due to the presence of the metal NPs or ions released from the NPs? The general consensus is that the mechanism of action is due to gold ions but in this case as part of an ionic complex, due to the fact that ionic gold is not as stable as ionic silver. It has been proposed that Au^+^ and Au^3+^ are the ionic forms predominantly responsible, with Au^+^ studied more extensively than Au^3+^. Au^+^ complexed with phosphine and n-heterocyclic carbenes has shown antimicrobial and antifungal activity. Au^3+^ organometallic complexes co-ordinated through Au-C or Au-N bonds interact with biological thiol groups found in bacterial enzymes which are essential for cell function [51]. Bacterial growth inhibition tests carried out by Dasari et al. revealed that both Au^+^ and Au^3+^ inhibit bacterial growth as a function of concentration. Further tests revealed that Au^3+^ is the more dominant ion as the bacterial inhibition significantly reduced with gradual removal of Au^3+^ ions. The cytotoxicity of Au^3+^ increases with concentration, posing possible problems *in vivo* [52].

### 1.5. Applications

Blaser likens the use of antibiotics to a ‘four-edged sword’ [53]. The initial use of antibiotics benefited both individuals and communities by fighting and preventing the spread of infection. The other two ‘edges’ transpired as antibiotic resistance became apparent in communities as well as the emergence that antibiotic use can alter ‘good’ bacteria which is essential to an individual’s microbiome. Van Boeckel et al. conducted a study of global antibiotic consumption in the period from 2000 to 2010 [54]. Their results revealed a 36% global increase in the use of antibiotics in the ten-year time period. An increasing global trend has been noted in the use of ‘last-resort’ polymixin antibiotics. This is believed to reflect the growth-rate of drug-resistant bacteria [55]. Bacterial evolution and inappropriate use of drugs contribute greatly to the development of said drug-resistant bacteria.

Cross-linked chitosan membranes with antimicrobial properties are possible candidates in the development of simple, low-cost, antimicrobial wound dressings. Previous studies have reported the development of chitosan wound dressings which show particular promise when reinforced by other natural materials [56,57]. For example, Zhao et al. found that electrospun chitosan/sericin composites effectively inhibit the growth of Gram-positive and Gram-negative bacteria by means of a simple colony-counting technique [58]. There is an urgent need for the development of such materials in our modern, antibiotic-dependant society which has arisen as a result of the emergence of drug-resistant bacteria. The aim of this present study is to investigate the structural changes induced on a chitosan network upon cross-linking with a siloxane network along with the inclusion of Ag and Au NPs and to look at the influence cross-linking may have on the antimicrobial properties of these composite materials.

## 2. Materials and methods

### 2.1. Synthesis of hydrogels and thin films

All chemicals were supplied by Sigma Aldrich, Ireland. The plain chitosan hydrogel and siloxane cross-linked interpenetrating polymer network (Chi-TEOS IPN) were synthesised by a cationic polymerisation technique adapted from [10] and as described in [4] utilising low molecular weight chitosan. Chi-TEOS IPNs containing Ag and Au NPs were prepared at a 0.02% concentration (0.02 mgmL^−1^ at 10 vol%). All samples were formed by a drop casting technique which involved drop casting a volume of 250 μL onto a hydrophilic glass surface with a surface area of 15 x 15 mm and allowing the sample to dry at 40 °C. Corning® plain microscope slides were employed in all experiments. This method could be easily modified according to the requirements for specific sample preparation.

### 2.2. Spectroscopic analysis

The Ag and Au NPs were analysed by UV-vis spectroscopy to confirm the dimensions of the particles; this was carried out on a Shimadzu UV-2401PC UV-vis spectrometer. Infrared analysis was carried out on free-standing thin film samples using a Bruker Alpha Fourier transform infrared spectrometer in attenuated total reflection mode which utilises a platinum-diamond crystal. A Renishaw inVia confocal Raman microscope was used for Raman analysis on thin film samples immobilised on glass slides. The structural differences between the plain chitosan and the hybrid cross-linked networks were investigated.

### 2.3. Microscopy

Scanning electron microscopy (SEM) analysis was performed using a FEI Quanta 650 FEG High Resolution scanning electron microscope. Typical beam energies were in the range of 10–20 kV. SEM analysis allowed for membrane thickness measurements and topological investigation. Transmission electron microscopy (TEM) was employed to image and measure Ag and Au NPs in the Chi-TEOS IPN membrane; these measurements were carried out on a JEOL 2100 High Resolution TEM, operated at 200 kV in bright field mode using a Gatan double tilt holder.

### 2.4. Surface and mechanical analysis

Surface roughness analysis was carried out using a KLA Tencor P15 profilometer, for further investigation into topological quality; this was done at a scanning rate of 20 μms^−1^ and a scanning distance of 1000 μm. The same test conditions were employed as in the preceding study [28] – an Instron 5565 universal testing machine was used to carry out tensile strength tests on the thin film membranes with rectangular geometry of 10 x 40 mm. Test conditions at room temperature included a load of 5 kN, clamp speed of 0.5 mm/min and data rate of 10 pts/s. The thin film strips were clamped with polydimethylsiloxane (PDMS) supports.

### 2.5. Antimicrobial tests

A crystal violet (CV) attachment assay [59] was carried out using Gram-negative bacteria – *E. coli*. The aim was to assess the affinity of bacterial samples for the various chitosan samples. Plain glass, chitosan, Chi-TEOS IPN, Chi-TEOS IPN-Ag and Chi-TEOS IPN-Au samples were all tested under aseptic conditions. Six slides of each sample were immersed in a six-well plate containing 2 x lysogeny broth (LB) growth medium, 2 x biological replicate 1 of bacterial sample in LB and 2 x biological replicate 2 of the same bacterial sample in LB. These were prepared at an initial optical density (OD) of 0.05 and stored at 37 °C for 24 hours, ideal growth conditions for *E. coli*. There were two controls in the form of the plain glass samples and the first two wells of each six-well plate bearing the LB growth medium but no bacteria. After 24 hours, the samples were thoroughly rinsed and stained with CV solution followed by rinsing in ethanol. The relative absorbance of all samples was measured allowing for a qualitative comparison. The absorbance was measured using a Tecan Genios plate reader with X Fluor 4 software on Microsoft Excel.

### 2.6. Release profile

An investigation into whether the Ag/Au NPs were capable of being released from the Chi-TEOS IPN network was carried out by immersing samples in relevant solutions and subsequently analysing the solutions using a Shimadzu UV-2401PC UV-vis spectrometer. The method involved immersing the cross-linked samples (Chi-TEOS IPN, Chi-TEOS IPN-Ag and Chi-TEOS IPN-Au) in 5 mL of 0.01 M phosphate buffered saline (PBS) solution in a series of six-well plates – six of each sample per plate, therefore three plates. The plates were then stored in an enclosed oven at 37 °C to replicate the growth environment for *E. coli* as described in section 2.4. Transmission spectra of the PBS solutions were measured at hourly intervals over a six-hour period, using the PBS with Chi-TEOS IPN samples immersed as the background correction. Transmission spectra were also recorded after 24 hours of storage at microbiological-replicate growth conditions. A few drops of 0.1 M NaCl were subsequently added to each solution to detect whether ions, rather than NPs, were released into the solution. Tests were also carried out in 0.1 M phosphate buffers at pH 2.5 and 11.5, to determine whether the release (if any) was pH-dependant due to the inherent pH-sensitivity of the chitosan network.

## 3. Results and discussion

### 3.1. Synthesis of hydrogels and thin films

**Figure 1.**
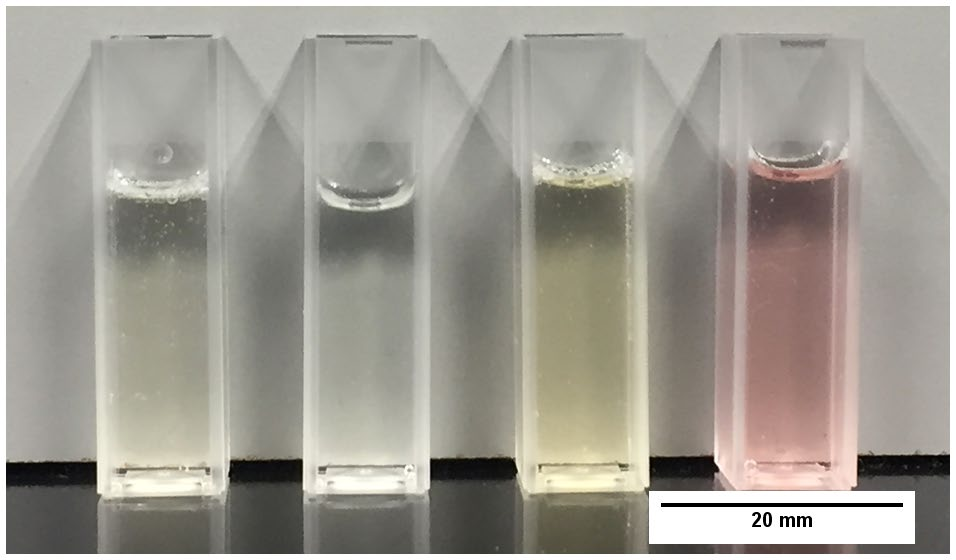
Chitosan composite hydrogels (L–R): plain chitosan, Chi-TEOS IPN, Chi-TEOS IPN-Ag and Chi-TEOS IPN-Au.

The chitosan hydrogels described in section 2.1 were prepared and deposited onto glass surfaces. It was important that the thin films were immobilised on a substrate in order to be able to perform the microbiological testing, because such samples need a solid support due to the rigorous washing and staining during the procedure for the CV attachment assay. This method was kept standard during other tests. Figure 1 displays the hydrogels; the yellow and pink appearance of the Chi-TEOS IPN-Ag/Au samples, respectively, is due to the surface plasmon resonance phenomenon which is characteristic of Ag and Au NPs.

### 3.2. Spectroscopic analysis

#### 3.2.1. UV-vis spectroscopy

**Figure 2.**
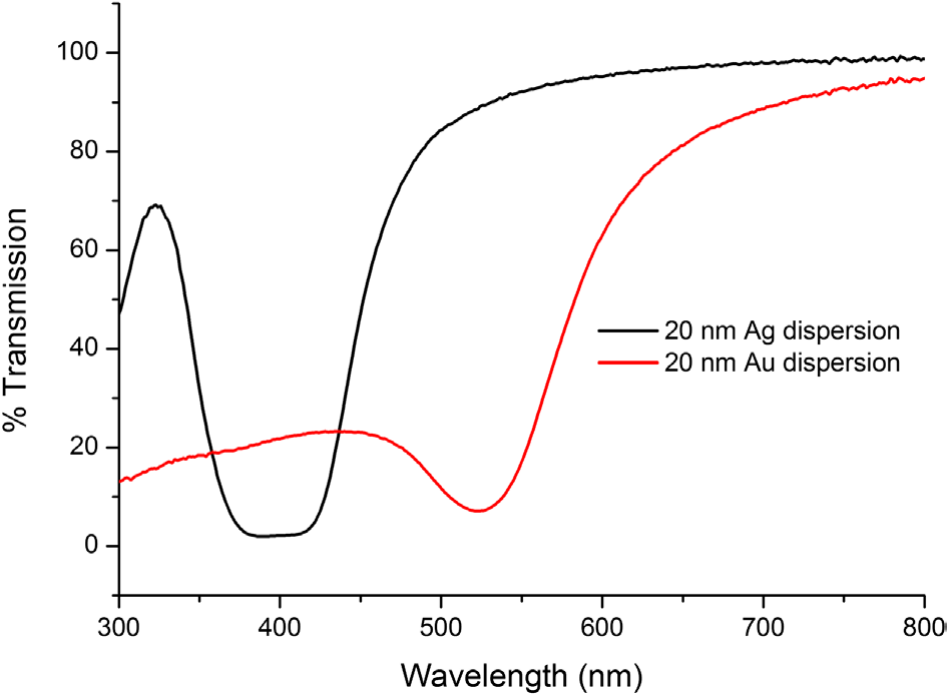
Transmittance spectra of Ag and Au dispersions.

Transmission measurements carried out on the metal NP dispersions by UV-vis spectroscopy revealed that the diameter of the Ag and Au NPs were 20 nm as the transmission wavelength corresponds with the literature values for the associated particle diameters. See Figure 2.

#### 3.2.2. Infrared (IR) spectroscopy

**Figure 3.**
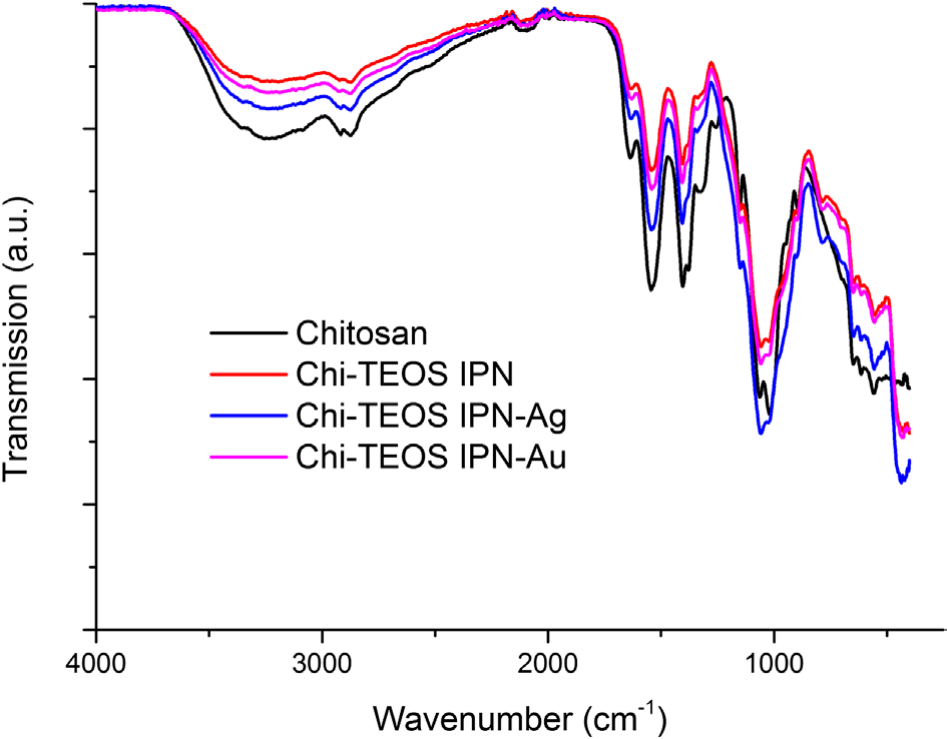
Infrared spectra of chitosan samples before and after cross-linking with siloxane polymer network and metal NPs.

The IR spectra for Chi-TEOS IPN-Ag and Chi-TEOS IPN-Au reveal that the same functional groups are present as in the Chi-TEOS IPN. The peak at 785 cm^−1^, which is not present for chitosan, is representative of deformations in amino (N-H) groups. A chitosan peak at 1250 cm^−1^ is lost at a frequency where methyl group stretches are usually found. This is probably due to an induced dipole disturbance to the structure caused by the presence of the cross-linked siloxane network. See Figure 3 for the associated IR spectrum. Full assignment of the IR spectra is available in [28].

#### 3.2.3. Raman spectroscopy

**Figure 4.**
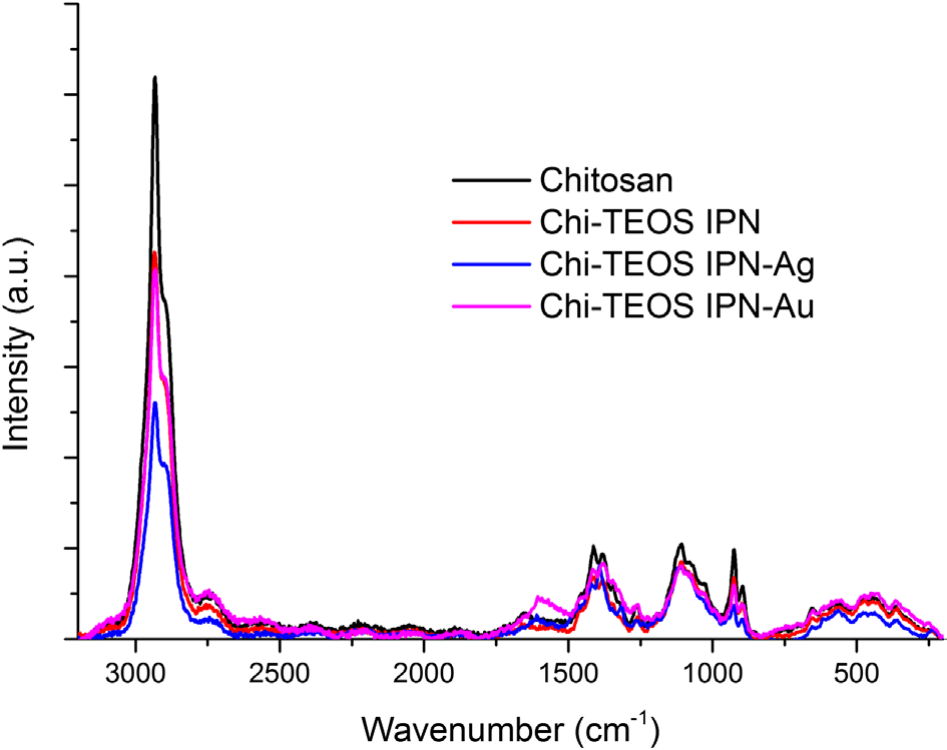
Raman spectra of chitosan samples before and after cross-linking with siloxane polymer network and metal NPs.

There appears to be very little disruption to the chitosan sample after cross-linking with the siloxane network and the metal NPs, according to the Raman data obtained, (see Figure 4). Again, this suggests that non-covalent, physically cross-linked linkages are being formed. The fact that no new peaks appear upon inclusion of the metal NPs highlights the lack of influence that the Ag/Au NPs have on the structure of the Chi-TEOS IPN, a trait which was also suggested from the IR spectra in Figure 3.

### 3.3. Microscopy

#### 3.3.1. SEM

**Figure 5.**
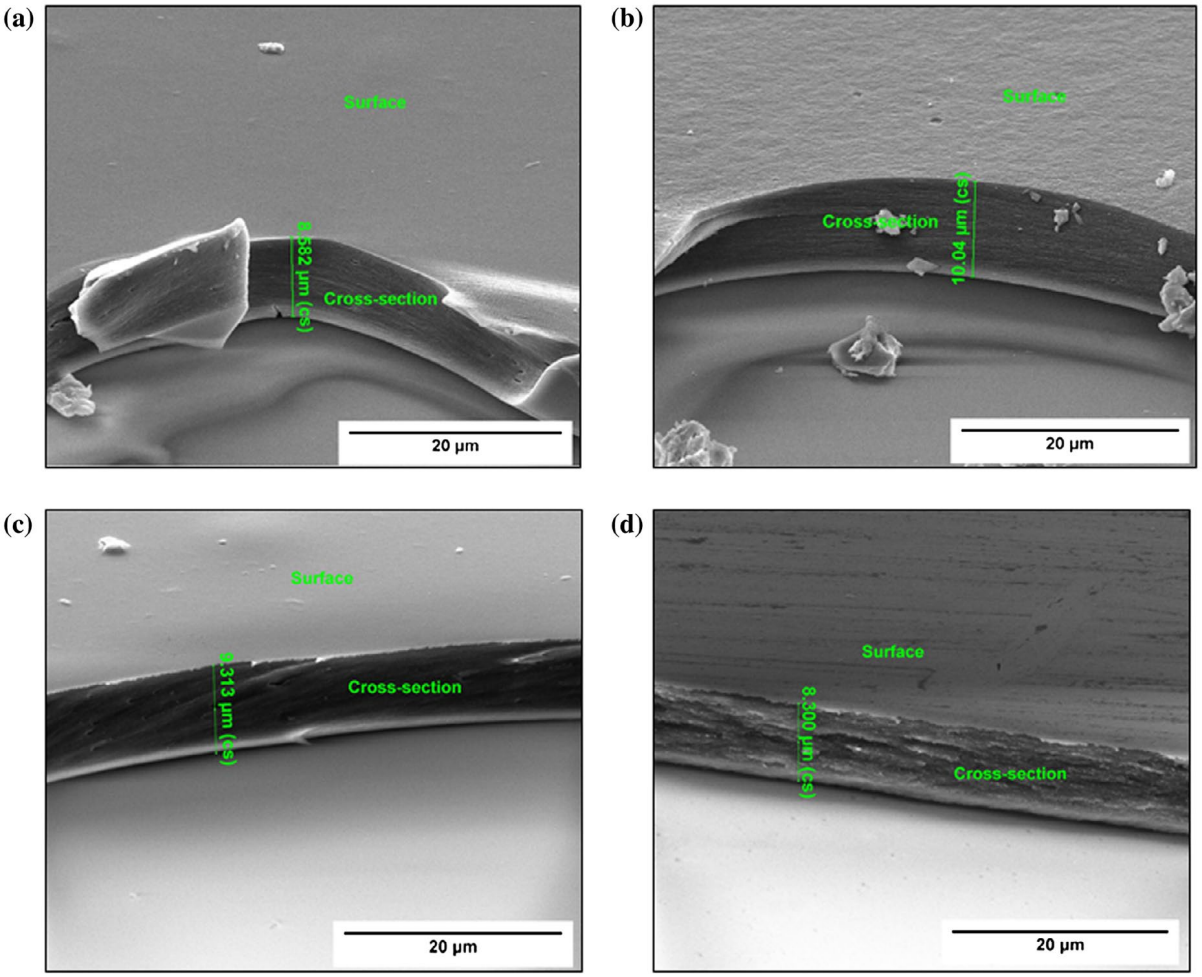
SEM images displaying the thickness and apparent surface roughness of the various chitosan samples: (a) plain chitosan, (b) Chi-TEOS IPN, (c) Chi-TEOS IPN-Ag and (d) Chi-TEOS IPN-Au.

SEM analysis revealed that the samples have a uniform thickness of 8–10 μm when prepared according to the method described in section 2.1. Thickness measurements yielded 8.58, 10.04, 9.31 and 8.30 μm for the chitosan, Chi-TEOS IPN, Chi-TEOS IPN-Ag and Chi-TEOS IPN-Au samples, respectively. Increased surface porosity was observed in the cross-linked samples; however, this is somewhat ambiguous due to the difficulties which arise in achieving contrast when imaging polymer samples. During these measurements it also became apparent that the thin film samples were not completely bound to the glass surface, but were perhaps remaining in place due to electrostatic interactions, possibly induced by the electron beam. The associated images can be seen in Figure 5.

#### 3.3.2. TEM

**Figure 6.**
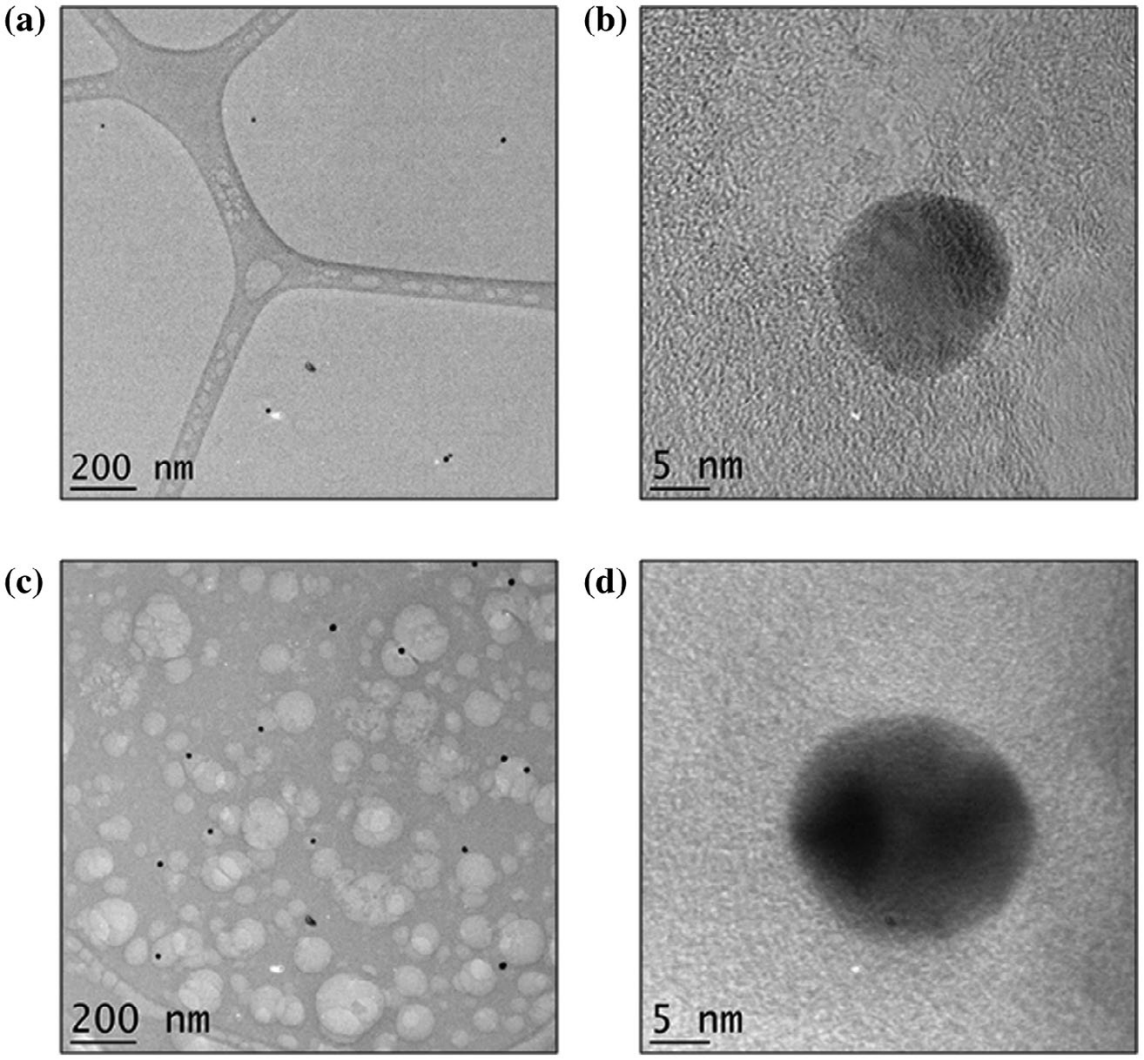
TEM images of (a) Ag NPs embedded in the Chi-TEOS IPN, (b) single Ag NP with one crystal facet observed, (c) Au NPs embedded in the Chi-TEOS IPN and (d) single multifaceted Au NP.

TEM analysis was chosen as a method for imaging the metal NPs in the IPN due to its high resolution as well as the fact that less charging occurs in comparison to SEM. The sample was prepared at a thickness of 200 nm by dropping < 0.3 μL of the 10 vol% Chi-TEOS IPN-Ag/Au hydrogel onto a lacy carbon TEM grid. Imaging was successful for both samples – see Figure 6. It became apparent that the Ag dispersion 6(a) is not as concentrated as the Au dispersion 6(c) by the number of particles counted per image; 38 Ag NPs were counted over ten images with an average particle size of 16 nm ± 25%, while for the Au dispersion 107 NPs were counted over 10 images and the average particle size was 19 nm ± 18%. A single Ag NP can be seen in Figure 6(b). The darker area shows the atomic scale crystal lattice, suggesting that the Ag NP is monocrystalline. Figure 6(d) shows an Au NP which seems to be polycrystalline due to the appearance of two dark areas, possibly representing two different crystal facets.

### 3.4. Surface and mechanical analysis

#### 3.4.1. Surface roughness measurements

Surface profiling was carried out in order to determine the roughness of the various samples, following the SEM observation that samples become more porous upon crosslinking. Scans were set at a speed of 20 μms^−1^ over a scanning distance of 1000 μm, with ten scans per sample. The results are outlined in Table 1. The surface roughness increases as the cross-linker density increases, a characteristic which was also suggested from analysis of the SEM images. Comparable results were seen between the membranes with NPs embedded, suggesting both the Ag and Au NPs act similarly within the membranes, causing the same subtle structural alterations. The greater percentage dispersity of the chitosan sample was due to a bubble on the sample surface which came in contact with the stylus.

**Table 1.** Surface roughness measurements of the various samples, showing an increased average surface roughness with increasing cross-linker density.

Sample	Average surface roughness (Å)	Dispersity (±%)
Chitosan	4588	250
Chi-TEOS IPN	6347	200
Chi-TEOS IPN-Ag	9174	209
Chi-TEOS IPN-Au	9722	196

#### 3.4.2. Tensile strength tests

**Figure 7.**
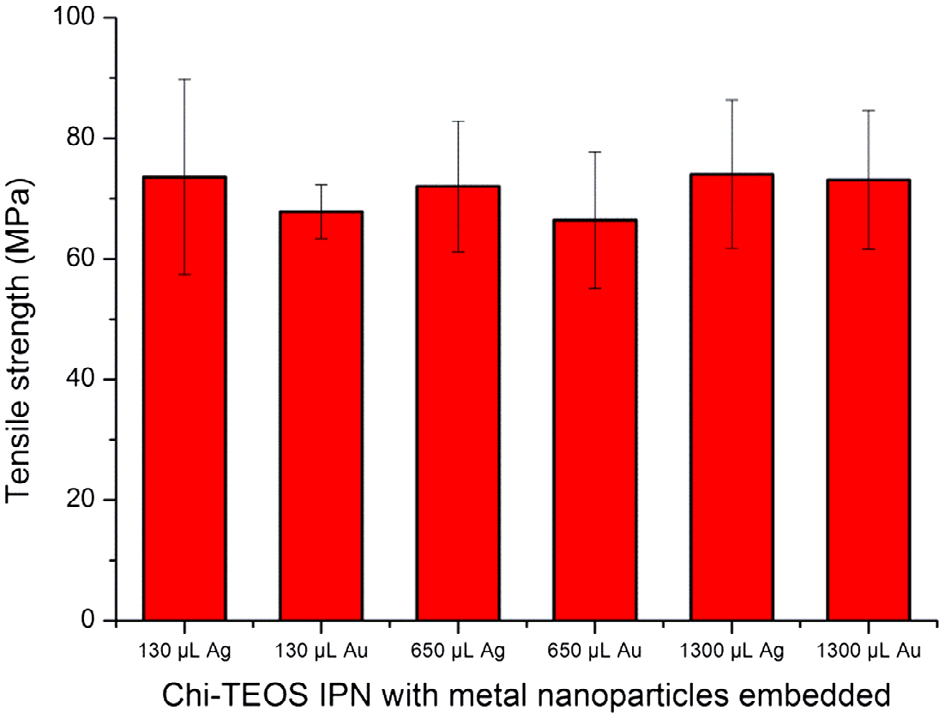
Tensile strength results with metal NPs embedded as a function of increasing concentration. Results are on a par with stresses reached by the Chi-TEOS IPN prior to the addition of NPs. No concentration dependence was observed.

The tests carried out investigated the effect of the presence of Ag and Au NPs with a diameter of 20 nm. Results showed that both NP types produced hybrid membranes capable of reaching mechanical stresses of up to 74 MPa, compared to 75 MPa ± 29% for the Chi-TEOS IPN [28]. This value was reached with the lowest concentration of metal NPs used and increasing this concentration made little or no change to the mechanical stress reached by the membranes. These results displayed low variance values, a favourable trait in terms of reproducibility of the samples. See Figure 7 for a graphical representation of the results as a function of increasing Ag/Au NP concentration: 130/ 650/ 1300 μL corresponding to 1/ 5/ 10 vol%, respectively.

Due to the dilute nature of the metal NP dispersions the Ag/Au NPs are present at a very low concentration. This may be the reason why the NPs do not appear to act as structure enhancers. The low concentration was also apparent in the TEM analysis. However, the low concentration of Ag/Au may still contribute towards enhancing the antimicrobial properties of a cross-linked chitosan membrane without causing appreciable degradation in mechanical strength – a property which would be highly desirable for certain types of anti-microbial membranes or drug delivery systems.

### 3.5. Crystal violet attachment assay

Results from the CV attachment assay with *E. coli* show that cross-linking contributes to the antimicrobial activity by reducing the attachment of bacteria to the surface by up to 80%. It is not known whether the siloxane cross-linker itself demonstrates antibacterial activity but, due to observations made throughout the tests, we suggest that the improved antibacterial activity of the Chi-TEOS IPN is due to the enhanced structural strength it imparts on the membranes. During the tests, the plain chitosan membranes were seen to completely detach from the glass substrate and disintegrate in the bacterial solution. Conversely, the cross-linked membranes, including those with embedded NPs, maintained their structural integrity and appeared to be almost unchanged after the tests were conducted. This again suggests that the cross-linkers enhance the strength of the membranes.

**Figure 8.**
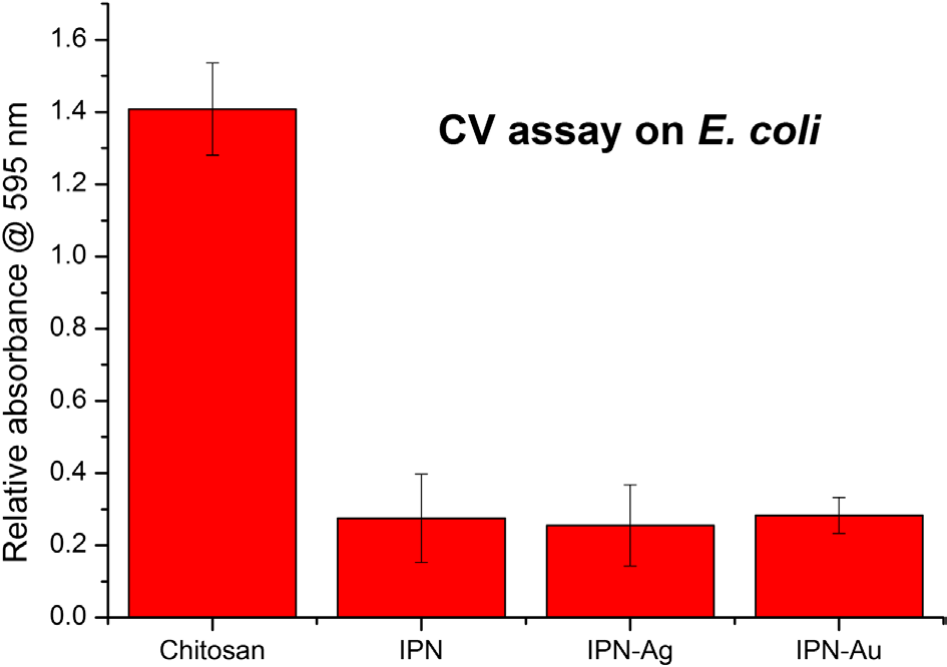
Relative absorbance values of chitosan, cross-linked Chi-TEOS IPN and Chi-TEOS IPN with Ag/Au NPs. The absorbance corresponds to the degree of bacterial attachment during the CV assay with *E. coli*. There is a significant reduction in attachment upon cross-linking with TEOS, while the addition of Ag/Au NPs does not contribute to antimicrobial activity.

It appears that the addition of Ag and Au NPs has no effect on the degree of bacterial attachment, indicating that the metal NPs do not contribute to the antimicrobial activity, perhaps because they are not being released from the membrane in neutral conditions. See Figure 8.

### 3.6. Release of Ag/Au NPs

**Figure 9.**
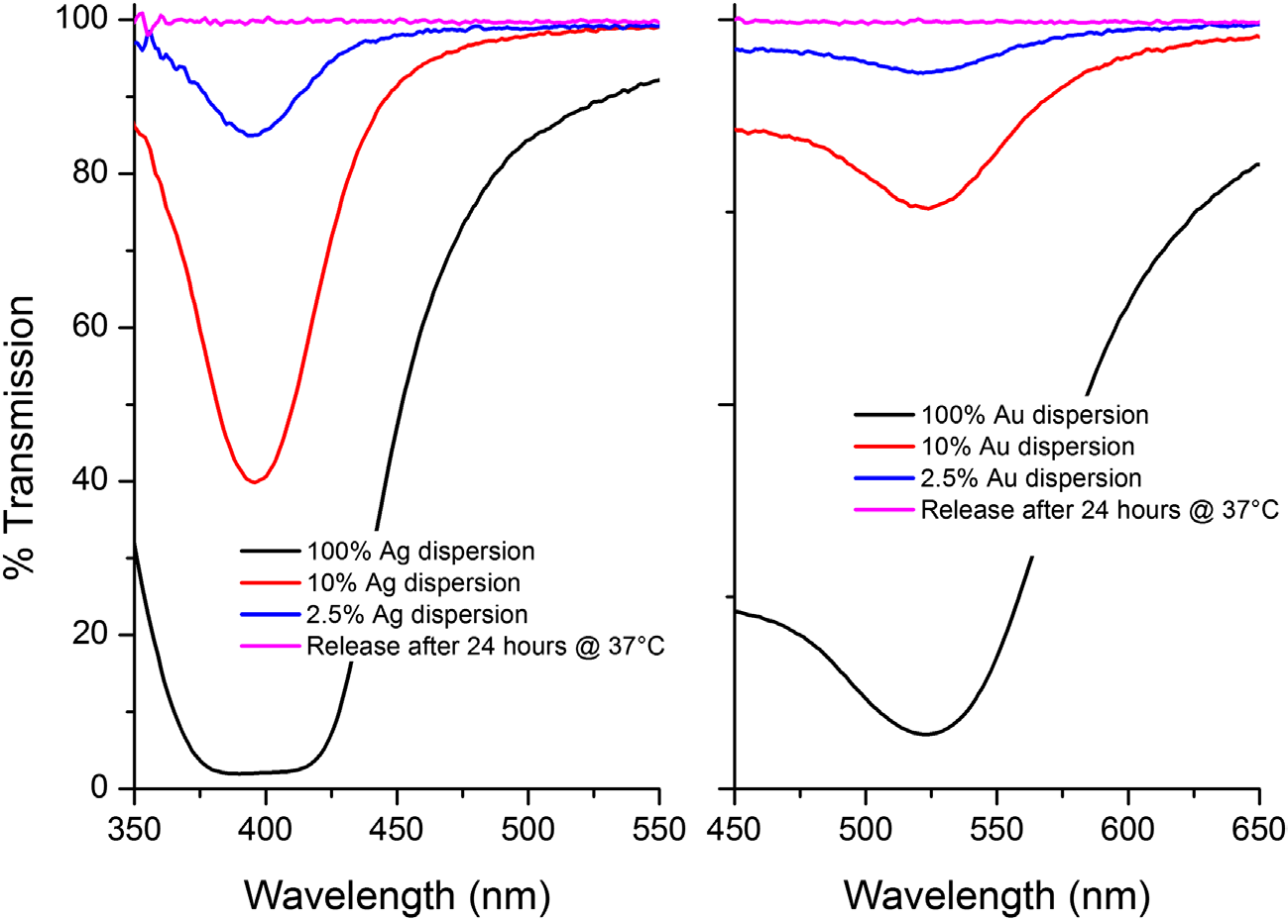
UV-vis spectra of Ag/Au NPs at a series of concentrations. 2.5% is the concentration of Ag/Au NPs present in each sample. This peak is not observed during the release tests meaning NPs are not being released.

Our measurements indicate that the metal NPs are not released in either NP or ionic form. If correct, this would indicate that their inclusion provides no additional contribution towards the antimicrobial activity, other than that which may arise from the enhanced mechanical properties alone. Figure 9 displays the UV-vis spectra of both Ag and Au NPs at a range of concentrations. The initial concentration of NPs employed was 2.5% and we estimate that the lower limit of detection of NPs from the UV-vis measurement was 0.25% (see Figure 11). However, when the PBS test solution was measured there was no peak observed, meaning that within experimental error there was no release of NPs into the solution, over the time allowed.

**Figure 10.**
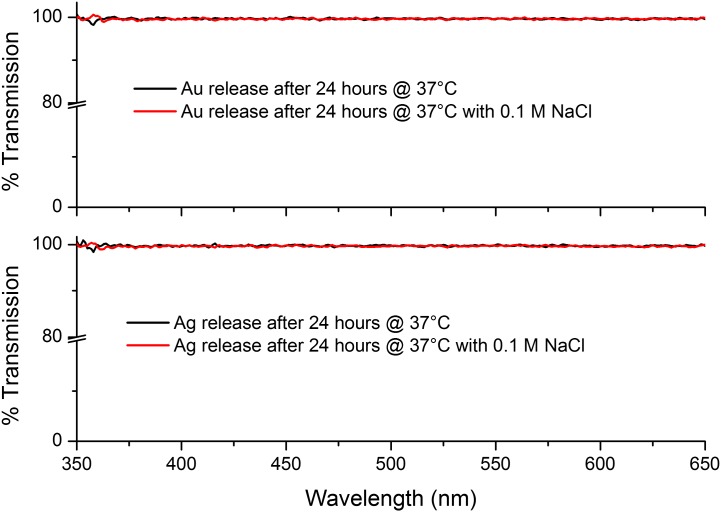
UV-vis spectra of the PBS solutions in combination with 0.1 M NaCl. Results indicate that Ag/Au ions are not being released as there is no change to the transmission spectrum.

As noted above, the possibility of ion release was investigated by adding a few drops of 0.1 M NaCl to the test’s PBS solution. If there were an appreciable amount of ions present the solution would turn cloudy due to the formation of AgCl/AuCl salts, which may be observable by eye or by a possible reduction in transmission. Neither of these changes were observed, suggesting that again, within the constraints of our measurements, no Ag/Au ion release was taking place (see Figure 10). Upon drying the samples the yellow/pink hue of the Ag/Au NPs was still observed, providing evidence that the NPs were still present in the Chi-TEOS IPN structure.

**Figure 11.**
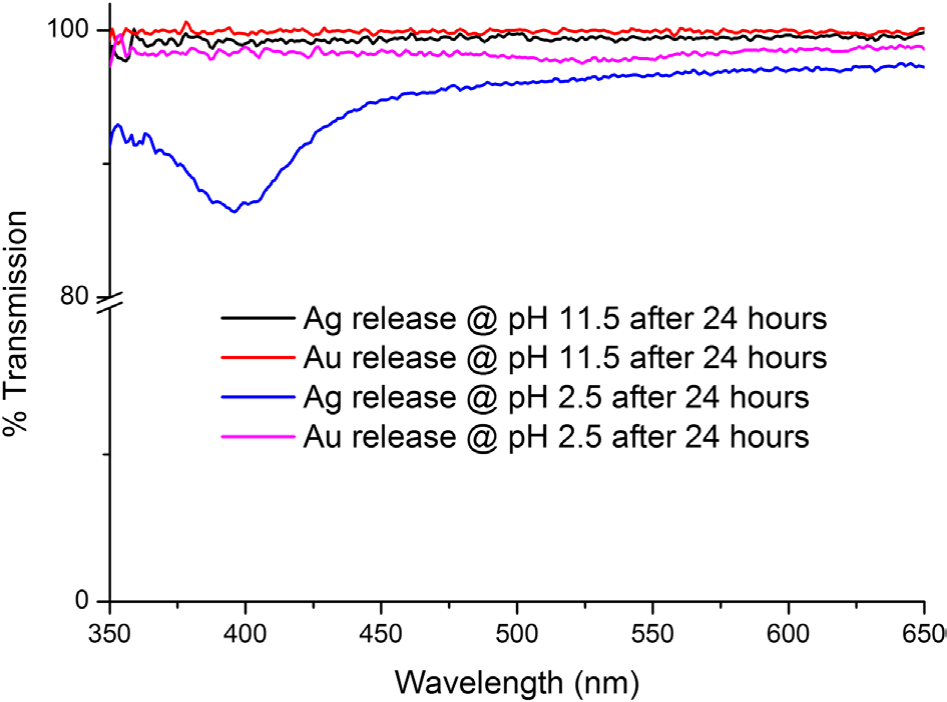
UV-vis spectra of Chi-TEOS IPN-Ag/Au samples being tested for release in basic and acidic conditions. The dips at about 395 and 525 nm indicate that the Ag and Au NPs are being released from the membrane in acidic conditions, respectively.

The release tests were carried out in phosphate buffers of pH 2.5 and 11.5 in order to investigate whether the Ag/Au NPs were being released in acidic/basic conditions. The IPNs might be expected to release the NPs at low pH as the chitosan network swells in acidic conditions due to the protonation of amino groups. The results of these release studies are shown in Figure 11.

From Figure 11, it is apparent that the Ag/Au NPs were released in acidic conditions, as expected. Thus, we conclude that the Ag/Au NPs can indeed be released from the membrane but the membrane must swell to allow for release. So, although not suitable for NP release under neutral conditions the cross-linked Chi-TEOS IPN with NPs embedded may be suitable for release in gastric conditions which are acidic in nature or in surface wounds which are often treated to deliberately create an acidic environment [60,61].

## 4. Conclusions

Chitosan along with silver and gold NPs are well known individually for their inherent antimicrobial properties [15,30,62]. The aim of this study was to investigate whether combining these materials would lead to any enhanced antimicrobial effects, induced by any structural or mechanical changes to the composite.

Our results demonstrate that the IPNs synthesised here show little evidence of any major chemical changes, although the inclusion of the metal NPs was found to influence the overall porosity of the IPN formed.

Under neutral conditions no evidence was found to support the hypothesis that the IPN could release either the metal NPs themselves, or ions derived from them; rather, the NPs were released under acidic conditions that would be more aligned to the conditions found in the digestive system, which would facilitate swelling of the chitosan part of the IPN. These observations account for the lack of additional antimicrobial activity arising from the presence of the metal NPs under the neutral conditions employed for the microbiological assay. It is therefore concluded that the metal NPs are simply physically confined within the IPN. With this in mind it is perhaps not so surprising that we found that the inclusion of the metal NPs also did not appreciably alter the mechanical properties of the films. What is perhaps surprising is that we have clearly identified additional mechanical strength and additional antimicrobial activity arising from the presence of the siloxane cross linker. In terms of the enhanced mechanical properties we clearly observe an increase in the robustness of the films in the bacteria solutions deployed in the assay, for those films containing the siloxane cross-linker. In terms of the degree of attachment of *E. coli* to the cross-linked IPN, we found that this was some ~80% less than the attachment of the same bacteria to the non-cross-linked chitosan.

This is a very positive result. Chitosan has been known to have antimicrobial effects on Gram-negative bacteria however our results show that providing additional mechanical strength to the chitosan network enhances this effect, in that the cross-linked membranes are much more stable in bacterial solution than the non-cross-linked chitosan membranes.

We suggest that our findings may stimulate the production of a range of chitosan/siloxane IPNs for a variety of biomedical applications such as wound dressings. In addition, we suggest that the release of metal NPs from these IPNs under acidic conditions may find application in the treatment of a range of disorders of the digestive system.

## Disclosure statement

No potential conflict of interest was reported by the authors.

## Funding

This work was supported by Science Foundation Ireland [grant numbers 11/PI/1117; 15/IA/3015; 13/ISCA/2846], by EU FP7 Program People: Marie Curie Actions Project ALBATROSS [grant number PIAPP-GA-2012-324449].
